# The Associations between Healthy Eating Patterns and Risk of Metabolic Dysfunction-Associated Steatotic Liver Disease: A Case–Control Study

**DOI:** 10.3390/nu16121956

**Published:** 2024-06-19

**Authors:** Xia Huang, Da Gan, Yahui Fan, Qihui Fu, Cong He, Wenjian Liu, Feng Li, Le Ma, Mingxu Wang, Wei Zhang

**Affiliations:** 1 The First Affiliated Hospital, Xi’an Jiaotong University Health Science Center, Xi’an 710061, China; ndyfy00382@ncu.edu.cn; 2 School of Public Health, Xi’an Jiaotong University Health Science Center, Xi’an 710061, China; fyh14042166@stu.xjtu.edu.cn (Y.F.); wangmx601@mail.xjtu.edu.cn (M.W.); 3 Jiangxi Medicine Academy of Nutrition and Health Management, The First Affiliated Hospital, Jiangxi Medical College, Nanchang University, Nanchang 330006, China; ganda@ncu.edu.cn (D.G.); fuqihui@email.ncu.edu.cn (Q.F.); ndyfy10076@ncu.edu.cn (W.L.); montanezd@email.phoenix.edu (F.L.); 4 Department of Gastroenterology, The First Affiliated Hospital, Jiangxi Medical College, Nanchang University, Nanchang 330006, China; ndydy04758@ncu.edu.cn; 5 Key Laboratory for Disease Prevention and Control and Health Promotion of Shaanxi Province, Xi’an 710061, China; 6 Key Laboratory of Environment and Genes Related to Diseases (Xi’an Jiaotong University), Ministry of Education of China, Xi’an 710061, China

**Keywords:** metabolic dysfunction-associated steatotic liver disease, Alternate Healthy Eating Index, Dietary Approaches to Stop Hypertension, Alternative Mediterranean Diet, vegetables, whole grain

## Abstract

Background: Although several epidemiological studies have identified an inverse association between healthy dietary patterns and metabolic dysfunction-associated steatotic liver disease (MASLD)/non-alcoholic fatty liver disease (NAFLD), little is known about the contribution of the food component to MASLD risk and the association between dietary patterns and severity of MASLD. This study aimed to investigate the association between healthy eating patterns and MASLD risk and severity of MASLD. Methods: A case–control study including 228 patients diagnosed with MASLD and 228 controls was conducted. The modified Alternate Healthy Eating Index (AHEI), Dietary Approaches to Stop Hypertension (DASH) score, and Alternative Mediterranean Diet (AMED) score were evaluated based on information collected via a validated food-frequency questionnaire. MASLD was confirmed if participants presented with ultrasound-diagnosed fatty liver diseases along with at least one of five cardiometabolic risk factors and no other discernible cause. The logistic regression models were applied to estimate the odds ratio (OR) and 95% confidence interval (95% CI) of MASLD for dietary scores. Results: Compared with participants in the lowest tertile, those in the highest tertile of AHEI had a 60% reduced risk of MASLD (OR: 0.40; 95% CI: 0.25–0.66). Similar associations were also observed for DASH and AMED, with ORs comparing extreme tertiles of 0.38 (95% CI: 0.22–0.66) and 0.46 (95% CI: 0.28–0.73), respectively. Further Stratified analysis revealed that the inverse associations between AHEI and DASH with MASLD risks were stronger among women than men, and the inverse associations between AMED and MASLD risks were more pronounced among participants with normal weight (OR: 0.22; 95% CI: 0.09–0.49). For components within the dietary score, every one-point increase in vegetable score and whole grain score within the AHEI was associated with an 11% (95% CI: 5–16%) and a 6% (95% CI: 0–12%) lower MASLD risk, respectively. Similar inverse associations with those scores were observed for the DASH and AMED. Conclusion: Greater adherence to healthy eating patterns was associated with reduced risk of MASLD, with vegetables and whole grains predominately contributing to these associations. These findings suggested that healthy eating patterns should be recommended for the prevention of MASLD.

## 1. Introduction

Metabolic dysfunction-associated steatotic liver disease (MASLD)/non-alcoholic fatty liver disease (NAFLD) is the leading cause of liver-related morbidity and mortality, affecting 32.4% of people in the world [1,2]. In China, the national prevalence of MASLD has increased rapidly, rising from 25.4% in 2008–2010 to 32.3% in 2015–2018 [3]. Moreover, the number of MASLD patients is projected to increase from 246.37 million cases in 2016 to 314.58 million cases in 2030 in the Chinese population [4]. MASLD is the primary cause of cirrhosis and hepatocellular carcinoma, which are irreversible with current therapies and can eventually result in premature death [5,6]. Therefore, identifying modifiable risk factors for MASLD is crucial for preventing liver-related diseases and extending lifespan.

As one of the important modifiable risk factors, diet has been demonstrated to play a significant role in the development of MASLD [7]. Previous studies have investigated the associations between individual food or nutrients and the risk of MASLD [8,9]. However, foods or nutrients were not consumed in isolation and each food or nutrient might interact synergistically, affecting human health in a collective manner [10]. Therefore, the dietary pattern, which was a combination of various foods and nutrients, could more closely reflect real-world dietary practices and the overall effect of diet [11,12]. Several epidemiological studies have examined the association between healthy eating patterns and MASLD/NAFLD, and most of these studies have revealed that greater adherence to healthy dietary patterns, as measured by the higher Alternate Healthy Eating Index (AHEI), the Dietary Approaches to Stop Hypertension (DASH), or the Alternative Mediterranean Diet (AMED) score, was significantly associated with lower risk of MASLD/NAFLD [13,14,15]. However, it is unclear whether increases in these dietary scores are associated with the severity of MASLD. Furthermore, it remains uncertain whether participants’ characteristics can modify the associations between healthy eating patterns and MASLD risk [14,15] given that both dietary choices and the prevalence of MASLD varied among participants with different characteristics [16]. Finally, debate continues regarding which components within dietary patterns contributed predominantly to the associations between dietary patterns and MASLD risk [15,17].

Therefore, this study aimed to examine the associations between dietary patterns and both MASLD risk and the severity of MASLD, and whether associations between dietary patterns and MASLD risk vary among participants with different characteristics. Additionally, the individual relationships between each component within dietary patterns and MASLD risk were examined.

## 2. Materials and Methods

### 2.1. Study Population

Potential participants were consecutively recruited from the physical examination center by posting advertisements and flyers between May 2023 and December 2023. Participants were eligible for inclusion as cases if they were older than 18 years and clinically diagnosed with MASLD. According to the criteria proposed by the Chinese Society of Hepatology of the Chinese Medical Association, a diagnosis of fatty liver disease was given when abdominal ultrasonography revealed liver brightness and diffusely echogenic changes in the liver [18]. Patients with ultrasound-diagnosed fatty liver diseases and restricted alcohol consumption (≤30 g/day for men or ≤20 g/day for women) were defined as having metabolic-associated fatty liver disease (MASLD) if they presented with at least one of the following cardiometabolic risk factors with no other discernible cause: (1) BMI ≥ 23 kg/m^2^; (2) fasting serum glucose ≥ 5.6 mmol/L or type 2 diabetes or treatment for type 2 diabetes; (3) blood pressure ≥ 130/85 mmHg or antihypertensive drug treatment; (4) plasma triglycerides ≥ 1.70 mmol/L or lipid-lowering treatment; (5) plasma HDL ≤ 1.0 mmol/L for men and ≤1.3 mmol/L for women or lipid-lowering treatment [1]. The severity of MASLD was determined according to the fibrosis-4 (FIB-4) index used by the Chinese Society of Hepatology of the Chinese Medical Association [19]. Each MASLD case underwent physical examination, liver function tests, and platelet counts, and was diagnosed with either metabolic-dysfunction associated steatotic liver (MASL) or hepatic fibrosis. Experienced sonographers conducted the abdominal ultrasonography using a specific ultrasonic equipment (LOGIQ S7, GE Ultrasound, Seongnam-si, Republic of Korea).

A total of 285 MASLD cases were recruited and all of them filled out a questionnaire and gave anthropometric measurements and fasting blood samples. MASLD cases were excluded if they were pregnant or lactating (*n* = 5), had difficulty communicating (*n* = 7), had cancer or an unstable chronic disease (*n* = 12), had missing information on dietary intake or covariates (*n* = 28), or reported implausible energy intake (<500 kcal/day or >3500 kcal/day) (*n* = 5). Finally, 228 MASLD cases were included in the current analysis. Among the included MASLD cases, 193 subjects were diagnosed with MASL while 35 were diagnosed with hepatic fibrosis. Employing the same exclusion criteria, an equal number of controls (*n* = 228) was randomly selected from age- and gender-matched adults who did not have MASLD during the same period. The MASLD cases and controls were recruited from the same physical examination center. Both groups were attracted to participate in this study through advertisements and flyers.

This study complied with the Helsinki Declaration guidelines and the protocol was approved by the Ethics Committee of First Affiliated Hospital of Nanchang University (No. IIT2023-146). All participants read and signed an informed consent form before data collection.

### 2.2. Assessment of Dietary Intake

Dietary intakes were evaluated using a validated food frequency questionnaire (FFQ) comprising 110 food items, which has been shown to have reasonable reproducibility and validity [20]. These food items covered the majority of commonly consumed foods, and each item was accompanied by a colorful food photography atlas with different portion sizes. Participants were asked to recall how often, on average (never to >6 times per day), they had consumed each food with standard serving sizes during the past year. Based on the data from the FFQ and the latest edition of the Chinese Food Composition Tables, dietary energy intake and daily nutrients were calculated. Intakes of each kind of food and nutrients were energy-adjusted using the nutrient density method, standardizing to 2000 kcal.

Three dietary quality scores (the modified AHEI, DASH, and AMED scores) were calculated to measure adherence to different dietary patterns [14,21,22,23,24]. The modified AHEI score consists of 10 components and the total score ranges from 0 to 100. The modified DASH includes 9 components, with the total score ranging from 9 to 45. The modified AMED includes 8 components and the total score ranges from 8 to 40. Higher scores indicate greater adherence to the dietary pattern. The details of the scoring criteria for each dietary score are presented in Table S1.

### 2.3. Assessment of Covariates

A structured questionnaire was utilized to collect information on demography, socioeconomic status, lifestyle, and medical history, including age, sex, education level, monthly household income, marriage, physical activity, smoking, and history of hypertension and type 2 diabetes. Physical activity was assessed using the long form of the International Physical Activity Questionnaire, and the weekly consumed metabolic equivalents of task were calculated [25]. Heights and weights were measured with participants barefoot. Body mass index (BMI) was calculated as weight (kg) divided by squared height (m^2^).

### 2.4. Statistical Analysis

In descriptive analyses, continuous variables were presented as mean (standard deviation) for normally distributed variables, median (interquartile range) for skewed variables, and frequency (percentage) for categorical variables. Comparisons between participants with and without MASLD were performed using the paired Student’s *t*-test for continuous variables with normal distribution, paired Wilcoxon signed-rank test for continuous variables with skewed distribution, and McNemar test for categorical variables. The relationship between each dietary score was estimated by calculating Pearson’s correlation coefficients.

Conditional logistic regression models were applied to estimate odds ratios (OR) and 95% confidence intervals (CI) for the associations of the dietary score tertiles with MASLD risk. The multivariable model was adjusted for age (continuous), education level (high school and below or college and above), monthly household income (<7000, ≥7000 yuan/capita), marriage (single/divorced/widowed or married/living together), physical activity (<21, ≥21 metabolic equivalents of task-hours/week), and smoking (yes or no). To remove the influence of confounding by total energy intake, the fully adjusted model was additionally adjusted for total energy intake (continuous) on the basis of the multivariable model. Tests for trends were conducted by assigning a median value to each tertile of dietary score as a continuous variable. We also explored the degree of reduced risk for MASLD associated with per 20-percentile increase in each dietary score (20 points for AHEI, 9 points for DASH, and 8 points for AMED). Ordinal logistic regression was also conducted to explore the associations between these dietary scores and the severity of MASLD.

Restricted cubic spline regressions with three knots were used to investigate the dose–response relationships between 3 dietary scores and MASLD risk. To test for non-linearity, the likelihood ratio test was conducted by comparing the model with the linear and cubic spline terms in the model with only the linear term.

To determine whether the associations between dietary score tertiles and prevalence of MASLD were modified by age, sex, BMI, education level, monthly household income, physical activity, smoking, or total energy intake, stratified analyses were performed using unconditional logistic regression models. Interactions between these factors and 3 dietary scores were examined using a likelihood ratio test comparing models with and without the multiplicative interaction terms.

For secondary analyses, to explore which component scores among the 3 dietary scores contributed significantly to the associations between dietary scores and MASLD risk, we examined the associations between individual component scores of the 3 dietary scores and MASLD risk. Several sensitivity analyses were performed to test the robustness of our findings: (1) utilizing the scoring criteria from the original edition of AHEI (Table S2) to recalculate AHEI scores, (2) evaluating the influence of additional adjustment for hyperlipemia, (3) restricting the analyses to participants without type 2 diabetes, (4) restricting the analyses to participants without hypertension, (5) assessing the influence of further adjustment for alcohol consumption, (6) excluding participants who were single/divorced/widowed, (7) restricting the analyses to newly-diagnosed MASLD patients, (8) investigating the associations between dietary scores and metabolic syndrome-related indicators [26].

All statistical analyses were conducted with Stata/SE 15.0 (StataCorp, College Station, TX, USA). A *p*-value of less than 0.05 (two-sided) indicated statistical significance.

## 3. Results

Table 1 presents the characteristics of MASLD cases and controls. The majority of MASLD participants (77.6%) in our study were newly diagnosed. Compared with the controls, the MASLD cases had higher BMI, triglycerides, low-density lipoprotein cholesterol, total cholesterol, fasting plasma glucose, alanine aminotransferase, aspartate aminotransferase, and total energy intake, and lower levels of AHEI scores, DASH scores, AMED scores, and high-density lipoprotein cholesterol. The prevalence of hypertension was also higher in MASLD cases than in controls. Individual dietary patterns were correlated with each other, with Pearson’s correlation coefficients ranging from 0.48 to 0.82.

**Figure 1:**
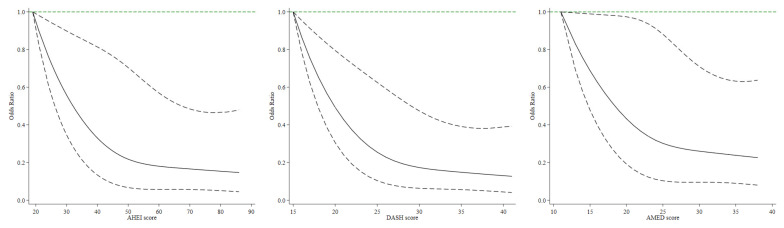
Restricted cubic spline analyses of associations between three dietary scores and risk of metabolic dysfunction-associated steatotic liver disease. Odds ratios were adjusted for age (continuous), education level (high school and below or college and above), monthly household income (<7000, ≥7000 yuan/capita), marriage (single/divorced/widowed or married/living together), physical activity (<21, ≥21 metabolic equivalents of task-hours/week), smoking (yes or no), and total energy intake (continuous).

Higher AHEI was associated with a lower risk of MASLD in the age-adjusted model (Table 2). After adjusting for multiple confounding factors in the multivariable model, the inverse associations remained largely unchanged. Additionally, after adjusting for total energy intake in the fully adjusted model, participants in the highest tertile of AHEI had a 60% reduced risk of MASLD (OR: 0.40; 95% CI: 0.25–0.66) compared with those in the lowest tertile. Similar associations with MASLD risk were also observed for DASH and AMED, with ORs comparing extreme tertiles of 0.38 (95% CI: 0.22–0.66) and 0.46 (95% CI: 0.28–0.73), respectively. Results from restricted cubic spline regressions indicated linear associations between the three dietary scores and the risk of MASLD (All *p*_linearity_ < 0.01) (Figure 1). Each 20-percentile increase in AHEI, DASH, and AMED score decreased the risk of MASLD by 43% (95% CI: 20–60%), 52% (95% CI: 29–67%), and 35% (95% CI: 12–52%), respectively.

In exploring the associations between dietary scores and severity of MASLD, results from ordinal logistic regressions revealed inverse associations with increased severity of MASLD for AHEI, DASH and AMED, with ORs comparing extreme tertiles of 0.44 (95% CI: 0.27–0.73) for AHEI, 0.49 (95% CI: 0.29–0.83) for DASH, 0.50 (95% CI: 0.31–0.82) for AMED, respectively (Table 3).

**Figure 2:**
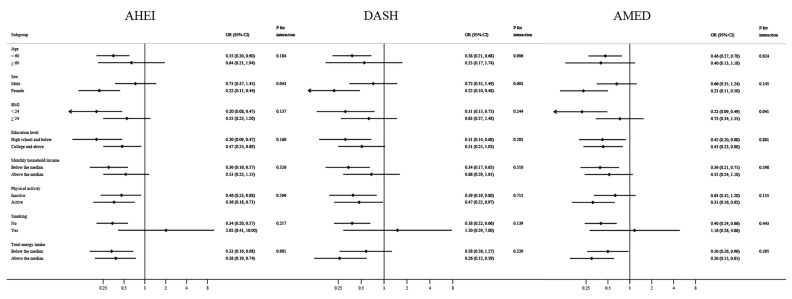
Stratified analyses of associations between three dietary scores and risk of metabolic dysfunction-associated steatotic liver disease (comparing the highest tertile of the dietary score with the lowest tertile). Odds ratios were adjusted for age (continuous), sex (male or female), education level (high school and below or college and above), monthly household income (<7000, ≥7000 yuan/capita), marriage (single/divorced/widowed or married/living together), physical activity (<21, ≥21 metabolic equivalents of task-hours/week), smoking (yes or no), and total energy intake (continuous). OR = odds ratio; CI = confidence interval.

The stratified analyses showed that most confounding factors did not modify the associations between dietary patterns and MASLD risk (Figure 2), with the exceptions of sex and BMI. The inverse associations between MASLD risk and AHEI and DASH were more pronounced among women (AHEI, OR: 0.22; 95% CI: 0.11–0.44; DASH, OR: 0.22; 95% CI: 0.10–0.48) than among men (AHEI, OR: 0.73; 95% CI: 0.37–1.45; DASH, OR: 0.72; 95% CI: 0.35–1.49) when comparing extreme tertiles. In addition, the reduction in MASLD risk in relation to AMED was greater among participants with normal weight (OR: 0.22; 95% CI: 0.09–0.49) compared to those who were overweight or obese (OR: 0.73; 95% CI: 0.34–1.55).

**Figure 3:**
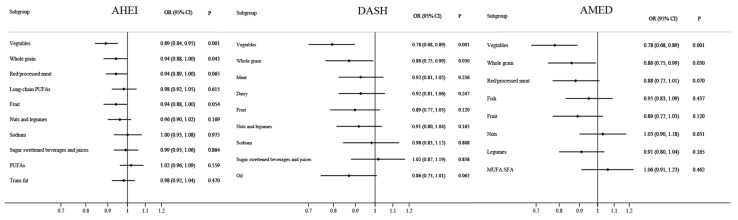
Associations between each component score within the three dietary scores and risk of metabolic dysfunction-associated steatotic liver disease. Odds ratios were adjusted for age (continuous), education level (high school and below or college and above), monthly household income (<7000, ≥7000 yuan/capita), marriage (single/divorced/widowed or married/living together), physical activity (<21, ≥21 metabolic equivalents of task-hours/week), smoking (yes or no), and total energy intake (continuous). OR = odds ratio; CI = confidence interval.

In estimating which component scores within the dietary scores contributed significantly to the associations between dietary scores and MASLD risk, the results revealed that every one-point increase in vegetable score and whole grain score within the AHEI decreased the risk of MASLD by 11% (95% CI: 5–16%) and 6% (95% CI: 0–12%), respectively (Figure 3). Similar inverse associations were observed for vegetable scores and whole grain scores within the DASH and AMED, with ORs of 0.78 (95% CI: 0.68–0.89) and 0.86 (95% CI: 0.75–0.99), respectively.

In sensitivity analyses, the associations across tertiles of the AHEI with risk of MASLD remained largely unchanged when AHEI was calculated using the original edition of scoring methods (Table S3). Similar associations between dietary scores and MASLD risk were observed after further adjustment for hyperlipidemia. Restricting the analyses to participants without type 2 diabetes or hypertension yielded similar results. Additional adjustments for alcohol consumption did not alter the findings. Results were similar after excluding participants who were single/divorced/widowed. The majority of MASLD participants (77.6%) in our study were newly diagnosed, and restricting the analyses to new-diagnosed MASLD patients did not materially change our findings. Compared with participants in the lowest tertile, those in the highest tertile of dietary scores were associated with a lower risk of high blood pressure, high triglyceride, and low high-density lipoprotein cholesterol, but not with the risk of hyperglycemia (Table S4).

## 4. Discussion

In the present study, higher AHEI, DASH, and AMED scores were inversely associated with MASLD risk and increased severity of MASLD. The inverse associations between AHEI and DASH and MASLD risk were more pronounced among women, and the inverse associations between AMED and MASLD risk were stronger among participants with normal weight. Furthermore, within the dietary patterns, vegetables and whole grains predominantly contributed to the inverse associations between dietary scores and MASLD risk. These findings support the protective role of high-quality dietary patterns in preventing MASLD.

Most observational studies have indicated that healthy eating patterns can lower the risk of MASLD/NAFLD. For example, findings from a cross-sectional study conducted in 584 outpatients with at least one cardiovascular risk factor in Italy revealed that the prevalence of liver steatosis decreased from 96.5% in patients who adhered strictly to the Mediterranean Diet to 71.4% in those with poor adherence to the Mediterranean Diet [27]. Results from randomized controlled trials also showed the beneficial effects of healthy eating pattern interventions in NAFLD patients. The 8-week interventions following the DASH diet improved BMI, liver enzymes, markers of insulin metabolism, and inflammatory markers [28], and the 12-week intervention following the Mediterranean diet reduced the weight of visceral fat [29]. In agreement with these findings, our results showed that a higher dietary quality was associated with a decreased risk of MASLD. Furthermore, this result was also supported by our finding, where the severity of illness decreased with the increment in dietary quality. Our findings suggest that these dietary patterns should be recommended for the prevention of MASLD.

The potential mechanisms underlying the decreased risk of MASLD’s association with high-quality dietary patterns involve the improvement of insulin resistance, inflammation, and weight gain, which were established risk factors for MASLD [30,31,32,33]. Healthy eating patterns were characterized by richness in high-quality carbohydrates, fresh fruits and vegetables, and legumes and nuts [34]. The nutrients abundant in healthy eating patterns like fiber, phytochemicals, and magnesium were effective in alleviating insulin resistance [35,36,37]. For instance, 8-week treatment with alginate and β-glucan in obese C57BL/6J mice improved glucose metabolism and insulin sensitivity by inactivating the c-Jun-*N*-terminal kinases signaling [35], and a 24-week intervention with phytochemicals in rats reduced blood glucose levels and enhanced insulin sensitization by upregulating Phosphoinositide 3-kinase, Phosphorylated Protein Kinase B, and Glucose transporter type 4 levels in the skeletal tissue [36]. Evidence from RCTs also demonstrated that interventions promoting a high-quality dietary pattern could improve insulin resistance [28]. Another mechanism contributing to the inverse associations between healthy dietary patterns and MASLD risk might involve the reduction of inflammation [38,39]. Inflammatory factors can prompt the development of MASLD by inducing liver injury and activating the inflammatory pathways that interfere with insulin signaling [31], and the abundant antioxidant and anti-inflammatory substances in high-quality dietary patterns could ameliorate inflammation [38,39]. A variety of cell experiments and animal studies have shown the positive effects of these substances against anti-inflammation, for example, lycopene lowered Tumor Necrosis Factor-alpha (TNF-α) activity in inflammation-stimulated human osteoblast cell lines and lutein-derived products decreased the concentrations of TNF-α and interleukin-6 (IL-6) in rats [40,41]. Furthermore, high-quality dietary patterns facilitated weight loss by reducing overall energy density, as they contain various nutrient-dense foods and limited amounts of energy-dense foods [42]. RCTs have shown the weight-loss effects of healthy eating pattern interventions in both healthy adults and MASLD patients [28,43]. Since obesity is an established risk factor for MASLD, high-quality dietary patterns might exert a beneficial influence on MASLD by contributing to weight loss [33].

Our stratified analyses showed that the inverse associations between adherence to healthy eating patterns and the prevalence of MASLD were stronger in women than in men. These findings were consistent with those of studies conducted by Xiao et al. and Sun et al. [14,15]. This might be because healthy eating patterns can lower the risk of MASLD by increasing concentrations of sex-hormone-binding globulin, and the inverse associations between sex-hormone-binding globulin and MASLD risk were reported to be stronger in women than in men [44,45]. In addition, our findings indicated that associations between healthy eating patterns and MASLD risk were more pronounced in participants with normal weight. This phenomenon might be attributed to the notion that healthy eating patterns can reduce the risk of MASLD by promoting weight loss, and the protective effects of healthy eating patterns on MASLD diminished in overweight individuals [28,33,43]. Further studies with large sample sizes are needed to replicate our findings.

Our study revealed that vegetables and whole grains predominately contributed to the inverse associations between dietary scores and the prevalence of MASLD. These findings align with previous research, which demonstrated that a higher intake of vegetables and whole grains (quintile 5 vs. quintile 1–4) can lead to 33% and 15% lower risk of MASLD, respectively [15]. Vegetables and whole grains might exert beneficial effects on MASLD by promoting weight loss, ameliorating inflammation, and improving insulin resistance [46,47,48]. In a study conducted by Kim et al. on C57BL/KsJ-db/db mice, a 5-week treatment of a whole grain diet significantly reduced the level of weight gain, pro-inflammatory cytokines, fasting, and postprandial blood glucose, insulin, and lipid accumulation in the liver compared to a normal diet [46]. These effects were attributed to the stimulation of the insulin receptor substrate 1/phosphoinositide 3-kinase pathway and the AMP-activated protein kinase/p38/Acetyl-CoA carboxylate pathway. In LDL receptor −/−, apolipoprotein B transgenic mice, feeding a diet rich in green and yellow vegetables for 16 weeks decreased body weight, plasma total cholesterol, and serum amyloid A (an index of inflammatory activity in mice) compared to mice fed a vegetable-free control diet [47]. Using obese Zucker rats as subjects, Ayoub et al. observed that substituting carbohydrates with purple vegetables in a high-fat diet resulted in a reduction in body weight gain, fasting plasma insulin levels, and Homeostatic Model Assessment of Insulin Resistance compared to the high-fat diet alone [48].

Several limitations of the study should be considered. First, given the observational nature of our study, the potential influence of unmeasured or residual confounding on the observed associations cannot be entirely eliminated even though we adjusted for a series of covariates. Second, information on dietary intake was collected using FFQ, and measurement errors were inevitable. However, this FFQ has been proven to have reasonable reproducibility and validity in comparison to dietary recall [20]. Third, MASLD was diagnosed using abdominal ultrasound but not liver biopsy, which is considered the established standard for MASLD diagnosis. Nevertheless, ultrasonography demonstrated comparable reliability and accuracy in the detection of moderate–severe fatty liver compared to liver biopsy [49]. MASLD diagnosed using ultrasound or liver biopsy showed similar associations with the risk of sub-clinical atherosclerosis [50]. Finally, the sample size in this study is relatively small and all participants were recruited in Nanchang City, which might limit the generalizability of our findings to the general population. Further studies with large sample sizes are needed to validate our findings in populations living in other areas.

## 5. Conclusions

In conclusion, higher adherence to AHEI, DASH, and AMED was associated with a reduced risk of MASLD and less severe MAFLD. Vegetables and whole grains predominantly contributed to the inverse associations between healthy eating patterns and MASLD risk. Furthermore, the inverse associations between AHEI and DASH and MASLD risk were more pronounced in women, and the inverse associations between AMED and MASLD risk were stronger in participants with normal weight. These findings suggest that healthy eating patterns should be recommended for the prevention of MASLD, with a specific emphasis on increased consumption of vegetables and whole grains.

## Figures and Tables

**Table 1:** The characteristics of participants with and without metabolic dysfunction-associated steatotic liver disease *.

Characteristics	No MASLD	MASLD	*p* Value
No. of participants	228	228	
Age, year	48.3 (14.2)	48.4 (13.9)	0.950
Men, *n* (%)	122 (53.5)	122 (53.5)	>0.999
College and above, *n* (%)	132 (57.9)	129 (56.6)	0.780
Monthly household income (≥7000 yuan/capita)	80 (35.1)	87 (38.2)	0.500
Married/living together, *n* (%)	201 (88.2)	205 (89.9)	0.550
Smokers, *n* (%)	30 (13.2)	35 (15.4)	0.500
Physical activity, MET hours/week	20.5 (4.6–49.5)	22.4 (6.6–44.8)	0.481
BMI, kg/m^2^	22.5 (3.1)	26.0 (3.2)	<0.001
Triglyceride, (mmol/L)	1.2 (0.9–1.5)	2.0 (1.5–2.8)	<0.001
Low-density lipoprotein cholesterol, (mmol/L)	2.6 (2.2–3.1)	2.9 (2.4–3.4)	<0.001
High-density lipoprotein cholesterol, (mmol/L)	1.3 (1.1–1.5)	1.1 (0.9–1.2)	<0.001
Total cholesterol, (mmol/L)	4.7 (4.2–5.4)	5.0 (4.5–5.8)	0.002
Fasting plasma glucose, (mmol/L)	4.8 (4.5–5.1)	5.0 (4.6–5.5)	<0.001
Alanine aminotransferase, (U/L)	16.4 (13.0–21.5)	26.9 (18.6–40.0)	<0.001
Aspartate aminotransferase, (U/L)	19.8 (17.4–23.0)	21.6 (18.0–27.0)	<0.001
Hypertension, *n* (%)	15 (6.6%)	37 (16.2%)	0.001
Type 2 diabetes, *n* (%)	6 (2.6%)	12 (5.3%)	0.150
Total energy intake, (kcal/day)	1723.0 (406.9)	1817.9 (480.6)	0.023
Alternate Healthy Eating Index score	51.9 (11.1)	48.0 (11.9)	<0.001
Dietary Approaches to Stop Hypertension score	27.7 (4.8)	26.3 (5.2)	0.004
Alternate Mediterranean Diet score	24.7 (4.9)	23.2 (5.1)	<0.001

* Continuous variables were presented as means (standard deviation) or median (interquartile range). Categorical variables were presented as frequency (percentage). MASLD = metabolic dysfunction-associated steatotic liver disease; BMI = body mass index; MET = metabolic equivalents of task.

**Table 2:** Odds ratios for metabolic dysfunction-associated steatotic liver disease based on tertiles of the three dietary scores.

Variables	Tertiles of Dietary Score			Per 20-Percentile Increase in the Dietary Score	*p*-Values for Trend
	1	2	3		
Alternate Healthy Eating Index score					
Median score	39	50	62		
No. of MASLD/No. of Non-MASLD	96/61	77/76	55/91		
Age-adjusted OR (95% CI)	1	0.60 (0.37 to 0.96)	0.39 (0.24 to 0.63)	0.55 (0.39 to 0.77)	<0.001
Multivariable-adjusted OR (95% CI) *	1	0.60 (0.37 to 0.97)	0.38 (0.23 to 0.62)	0.54 (0.38 to 0.76)	<0.001
Fully adjusted OR (95% CI) †	1	0.63 (0.39 to 1.03)	0.40 (0.25 to 0.66)	0.57 (0.40 to 0.80)	<0.001
Dietary Approaches to Stop Hypertension score					
Median score	22	27	33		
No. of MASLD/No. of Non-MASLD	91/63	75/87	62/78		
Age-adjusted OR (95% CI)	1	0.58 (0.36 to 0.94)	0.54 (0.33 to 0.87)	0.62 (0.45 to 0.87)	0.012
Multivariable-adjusted OR (95% CI) *	1	0.57 (0.35 to 0.93)	0.52 (0.32 to 0.86)	0.60 (0.42 to 0.86)	0.011
Fully adjusted OR (95% CI) †	1	0.50 (0.30 to 0.83)	0.38 (0.22 to 0.66)	0.48 (0.33 to 0.71)	0.001
Alternate Mediterranean Diet score					
Median score	19	23	29		
No. of MASLD/No. of Non-MASLD	91/60	74/68	63/100		
Age-adjusted OR (95% CI)	1	0.71 (0.43 to 1.15)	0.44 (0.28 to 0.70)	0.63 (0.47 to 0.85)	<0.001
Multivariable-adjusted OR (95% CI) *	1	0.71 (0.44 to 1.16)	0.43 (0.27 to 0.69)	0.63 (0.46 to 0.84)	<0.001
Fully adjusted OR (95% CI) †	1	0.75 (0.46 to 1.24)	0.46 (0.28 to 0.73)	0.65 (0.48 to 0.88)	0.001

* The multivariable-adjusted model was adjusted for age (continuous), education level (high school and below or college and above), monthly household income (<7000, ≥7000 yuan/capita), marriage (single/divorced/widowed or married/living together), physical activity (<21, ≥21 metabolic equivalents of task-hours/week), and smoking (yes or no). † The fully adjusted model was further adjusted for total energy intake (continuous). OR = odds ratio; CI = confidence interval.

**Table 3:** Odds ratios for increased severity of metabolic dysfunction-associated steatotic liver disease based on tertiles of the three dietary scores.

Variables	No. of Participants with Fibrosis/Participants with Fatty Liver/Participants without MASLD	Odds Ratio (95% CI) Based on Tertiles of Dietary Score			*p*-Values for Trend
		1	2	3	
Alternate Healthy Eating Index score	35/193/228	1	0.71 (0.44 to 1.14)	0.44 (0.27 to 0.73)	<0.001
Dietary Approaches to Stop Hypertension score	35/193/228	1	0.59 (0.36 to 0.95)	0.49 (0.29 to 0.83)	0.001
Alternate Mediterranean Diet score	35/193/228	1	0.91 (0.56 to 1.47)	0.50 (0.31 to 0.82)	0.001

Odds ratios were adjusted for age (continuous), sex (male or female), education level (high school and below or college and above), monthly household income (<7000, ≥7000 yuan/capita), marriage (single/divorced/widowed or married/living together), physical activity (<21, ≥21 metabolic equivalents of task-hours/week), smoking (yes or no), and total energy intake (continuous). MASLD = metabolic dysfunction-associated steatotic liver disease; CI = confidence interval.

## Data Availability

Data can be obtained from the corresponding author upon a reasonable request.

## Supplementary Materials

The following supporting information can be downloaded at: https://www.mdpi.com/article/10.3390/nu16121956/s1, Table S1: Criteria for scoring each component of three dietary scores. Table S2: Criteria for scoring each component of the original edition of Alternate Healthy Eating Index. Table S3: Sensitivity analyses for associations between dietary scores and metabolic dysfunction-associated steatotic liver disease. Table S4: Odds Ratios for metabolic syndrome-related indicators according to tertiles of the three dietary score.
