# Supplementary material for: The Associations between Healthy Eating Patterns and Risk of Metabolic Dysfunction-Associated Steatotic Liver Disease: A Case–Control Study
Source: Nutrients. 2024 Jun 19;16(12):1956. doi: 10.3390/nu16121956 (PMC11207114; doi:10.3390/nu16121956)
Supplement: Supplementary file 1 [file nutrients-16-01956-s001.zip › nutrients-2962897-supplementary.pdf]

Table S1. Criteria for scoring each component of three dietary scores.

Component	Alternate Healthy Eating Index- Criteria for minimum score of 0	Alternate Healthy Eating Index- Criteria for maximum score of 10	Dietary approaches to stop hypertension Criteria for minimum score of 1	Dietary approaches to stop hypertension Criteria for maximum score of 5	Alternate Mediterranean Diet Criteria for minimum score of 1	Alternate Mediterranean Diet Criteria for maximum score of 5
Vegetables (excluding potatoes), g/d	Lowest decile	Highest decile	Lowest quintile	Highest quintile	Lowest quintile	Highest quintile
Fruit, g/d	Lowest decile	Highest decile	Lowest quintile	Highest quintile	Lowest quintile	Highest quintile
Whole grains (including sweet potatoes), g/d	Lowest decile	Highest decile	Lowest quintile	Highest quintile	Lowest quintile	Highest quintile
Sugar-sweetened beverages and fruit juice, g/d	Highest decile	Lowest decile	Highest quintile	Lowest quintile		
Nuts and legumes, g/d	Lowest decile	Highest decile	Lowest quintile	Highest quintile		
Nuts, g/d	--	--			Lowest quintile	Highest quintile
Legumes, g/d	--	--			Lowest quintile	Highest quintile
Red and processed meat, g/d	Highest decile	Lowest decile			Highest quintile	Lowest quintile
Meat, g/d			Highest quintile	Lowest quintile		
Fish, g/d	--	--	--	--	Lowest quintile	Highest quintile
Fat (vegetable oil and animal oil), g/d			Highest quintile	Lowest quintile		
Dairy, g/d	--	--	Lowest quintile	Highest quintile		
Trans fat, % of energy	Highest decile	Lowest decile				
Long-chain (n-3) fats (EPA + DHA), mg/d	Lowest decile	Highest decile				
Poly-unsaturated fatty acids, % of energy	Lowest decile	Highest decile				

MUFA:SFA ratio	--	--			Lowest quintile	Highest quintile
Sodium, mg/d	Highest decile	Lowest decile	Highest quintile	Lowest quintile		
TOTAL	**0**	**100**	**9**	**45**	**8**	**40**

Table S2. Criteria for scoring each component of the original edition of Alternate Healthy Eating Index.

Component	Criteria for minimum score of 0	Criteria for maximum score of 10
Vegetables (excluding potatoes), servings/d	0	≥ 5
Fruit, servings/d	0	≥ 4
Whole grains (including sweet potatoes), servings/d		
Men	0	6
Women	0	5
Sugar-sweetened beverages and fruit juice, servings/d	≥ 1	0
Nuts and legumes, servings/d	0	≥ 1
Red and processed meat, servings/d	≥ 1.5	0
Trans fat, % of energy	≥ 4	≤ 0.5
Long-chain (n-3) fats (EPA + DHA), mg/d	0	250
Poly-unsaturated fatty acids, % of energy	≤ 2	≥ 10
Sodium, mg/d	Highest decile	Lowest decile

Vegetables: one serving = 80g; Frutis: one serving = 80g; Whole grains: one serving = 15g; Sugar-sweetened beverages and fruit juice: one serving = 224 g; Nuts: one serving = 28g; Legumes: one serving = 113g; Red and processed meat: one serving = 113g.

Table S3. Sensitivity analyses for associations between dietary scores and metabolic dysfunction-associated steatotic liver disease.

Variable	Odds ratio (95% CI) by tertiles of dietary score			P values for trend
	1	2	3	
Alternate Healthy Eating Index score				
Using another method to calculate score	1	0.81 (0.50 to 1.30)	0.46 (0.28 to 0.75)	0.002
Adjustment for hyperlipemia	1	0.72 (0.41 to 1.26)	0.43 (0.25 to 0.76)	0.003
Excluding participants with type 2 diabetes	1	0.62 (o.38 to 0.99)	0.41 (0.25 to 0.66)	< 0.001
Excluding participants with hypertension	1	0.68 (0.42 to 1.12)	0.42 (0.25 to 0.70)	0.001
Adjustment for alcohol consumption	1	0.63 (0.39 to 1.03)	0.40 (0.25 to 0.66)	< 0.001
Excluding participants who were single/divorced/widowed	1	0.56 (0.34 to 0.93)	0.33 (0.20 to 0.55)	< 0.001
Restricting analyses to newly diagnosed MASLD patients	1	0.61 (0.35 to 1.07)	0.45 (0.26 to 0.78)	0.005
Dietary approaches to stop hypertension score				
Adjustment for hyperlipemia	1	0.52 (0.29 to 0.94)	0.44 (0.23 to 0.85)	0.015
Excluding participants with type 2 diabetes	1	0.52 (0.32 to 0.84)	0.40 (0.24 to 0.69)	0.001
Excluding participants with hypertension	1	0.51 (0.31 to 0.84)	0.43 (0.25 to 0.75)	0.003
Adjustment for alcohol consumption	1	0.50 (0.30 to 0.82)	0.38 (0.22 to 0.66)	0.001
Excluding participants who were single/divorced/widowed	1	0.44 (0.27 to 0.73)	0.32 (0.18 to 0.55)	< 0.001
Restricting analyses to newly diagnosed MASLD patients	1	0.49 (0.27 to 0.86)	0.41 (0.22 to 0.77)	0.007
Alternate Mediterranean Diet score				

Adjustment for hyperlipemia	1	1.00 (0.57 to 1,77)	0.52 (0.29 to 0.92)	0.014
Excluding participants with type 2 diabetes	1	0.71 (0.44 to 1.15)	0.43 (0.26 to 0.69)	< 0.001
Excluding participants with hypertension	1	0.77 (0.47 to 1.27)	0.45 (0.28 to 0.75)	0.002
Adjustment for alcohol consumption	1	0.76 (0.46 to 1.25)	0.46 (0.28 to 0.73)	0.001
Excluding participants who were single/divorced/widowed	1	0.67 (0.40 to 1.11)	0.40 (0.25 to 0.66)	< 0.001
Restricting analyses to newly diagnosed MASLD patients	1	0.73 (0.42 to 1.25)	0.54 (0.32 to 0.90)	0.019

The model was adjusted for age (continuous), education (high school and below or college and above), monthly household income (< 7000, ≥ 7000 yuan/capita), marriage (single/divorced/widowed, married/living together), physical activity (< 20.5, ≥ 20.5 metabolic equivalents of task-hours/week), smoking (yes or no), total energy intake (continuous), and body mass index (continuous), history of hypertension (yes or no) and diabetes (yes or no), unless otherwise indicated.

CI=confidence interval.

Table S4. Odds Ratios for metabolic syndrome-related indicators according to tertiles of the three dietary score.

	No. of MASLD patient/ No. of non-MASLD patient	Odds ratio (95% CI) by tertiles of dietary score		P values for trend
		2	3	
Alternate Healthy Eating Index score				
High blood pressure	196/259	0.59 (0.36 to 0.97)	0.49 (0.29 to 0.81)	0.006
High triglyceride	228/227	0.80 (0.50 to 1.28)	0.55 (0.34 to 0.90)	0.016
Low high-density lipoprotein cholesterol	162/290	0.65 (0.40 to 1.04)	0.56 (0.35 to 0.90)	0.018
Hyperglycemia	74/372	1.34 (0.71 to 2.64)	0.78 (0.38 to 1.57)	0.412
Dietary approaches to stop hypertension score				
High blood pressure	196/259	0.59 (0.36 to 0.97)	0.53 (0.31 to 0.92)	0.021
High triglyceride	228/227	0.91 (0.57 to 1.44)	0.63 (0.37 to 1.05)	0.077
Low high-density lipoprotein cholesterol	162/290	0.91 (0.57 to 1.46)	0.51 (0.31 to 0.86)	0.011
Hyperglycemia	74/372	0.69 (0.37 to 1.31)	0.57 (0.28 to 1.16)	0.115
Alternate Mediterranean Diet score				
High blood pressure	196/259	0.56 (0.34 to 0.93)	0.44 (0.27 to 0.72)	0.002
High triglyceride	228/227	0.58 (0.36 to 0.94)	0.53 (0.33 to 0.85)	0.014
Low high-density lipoprotein cholesterol	162/290	0.56 (0.34 to 0.90)	0.59 (0.37 to 0.94)	0.047
Hyperglycemia	74/372	0.99 (0.52 to 1.91)	0.67 (0.35 to 1.30)	0.216

Odd ratios were adjusted for age (continuous), sex (male or female), education (high school and below or college and above), monthly household income (< 7000, ⩾ 7000 yuan/capita), marriage (single/divorced/widowed, married/living together), physical activity (< 21.0, ⩾ 21.0 metabolic equivalents of task-hours/week), smoking (yes or no), and total energy intake (continuous). OR=odds ratio; CI=confidence interval.
